# Acute Changes in Inflammatory Biomarker Levels in Recreational Runners Participating in a Marathon or Half-Marathon

**DOI:** 10.1186/s40798-016-0045-0

**Published:** 2016-03-02

**Authors:** Markus Niemelä, Päivikki Kangastupa, Onni Niemelä, Risto Bloigu, Tatu Juvonen

**Affiliations:** 1 Department of Surgery, Oulu University Hospital, P.O. Box 21, 90029 OYS Oulu, Finland; 2 Department of Laboratory Medicine and Medical Research Unit, Seinäjoki Central Hospital and University of Tampere, Hanneksenrinne 7, 60220 Seinäjoki, Finland; 3 Medical Informatics and Statistics Research Group, University of Oulu, Oulu, Finland

## Abstract

**Background:**

Strenuous physical activity activates the participant’s immune responses; however, few studies exist, observing exercise-induced simultaneous changes in mediators of inflammation.

**Methods:**

We examined individual responses in soluble urokinase-type plasminogen activator receptor (suPAR), a marker of immune activation, soluble endocytic receptor for haptoglobin-hemoglobin complexes (CD163), a marker of monocyte-macrophage activation, C-reactive protein (CRP), and pro- and anti-inflammatory cytokines from blood samples drawn at baseline, at 3- and 48-h post-races from recreational runners who successfully completed the marathon (199 ± 8 min, *n* = 4) or half-marathon (132 ± 4 min, *n* = 4) run. For comparisons, biomarkers reflecting muscle, heart, kidney, and liver functions were measured.

**Results:**

Significant 3-h post-race increases occurred in levels of suPAR (*p* < 0.01), CD163 (*p* < 0.05), white blood cells (*p* < 0.001), pro-inflammatory cytokines, interleukin-6 (IL-6) (*p* < 0.001), IL-8 (*p* < 0.05), and anti-inflammatory cytokine IL-10 (*p* < 0.05), whereas tumor necrosis factor-α (TNF-α) and transforming growth factor-β (TGF-β) remained relatively stable. Full-marathon running lead to more pronounced increases in suPAR, CD163, IL-8, and IL-10 than half-marathon running. In addition, 3-h post-race increases of all these parameters correlated significantly with changes in serum TNF-α and cortisol. The 48-h levels of serum suPAR and both pro- and anti-inflammatory cytokines had decreased to baseline levels, whereas CRP, a marker of acute phase response, increased in those with the most prominent IL-6 and IL-10 elevations in their preceding samples. The highest suPAR, CRP, IL-6, TNF-α, IL-10, and cortisol levels were noted in the individual with the most severe post-race fatigue.

**Conclusions:**

Prolonged running increases mediators of inflammation in an exercise-dose-dependent manner which should be considered in the assessment of health status of physically active individuals after recent acute bouts of strenuous exercise.

## Key Points

- Marathon and half-marathon running lead to distinct changes in mediators of inflammation in an exercise-dose-dependent manner.
- Alterations in the balance between mediators of inflammation may contribute to the post-race reactivity of the immune system and health status in individuals participating in such events.

## Background

Although physical activity is widely acknowledged to be an important part of a healthy lifestyle, the required doses of activity leading to beneficial health effects remain as an issue of controversy [1–4]. Long-distance running and associated competitive events are currently attracting ever-increasing numbers of participants, who present with significant differences in their training backgrounds. Consequently, marked inter-individual variation may also be expected to occur in the acute health effects brought about by such strenuous physical activity.

Several previous studies among marathon or ultramarathon runners have emphasized potential acute health threats related to vigorous exercise, including the development of muscle, heart, or kidney injury [5–7]. As yet, however, relatively little is known on simultaneous comparisons of individual responses in the mediators of inflammation and on the interactions between such responses and other biomarkers of organ health [8–11]. More information is also needed on the magnitude of changes in such biomarkers as a result of either marathon or half-marathon running by non-elite recreational runners.

Recent studies have indicated that IL-6, which is widely recognized as a pro-inflammatory cytokine [12], may also have a role as a myokine with anti-inflammatory properties [9, 11]. Previous researchers suggest that aerobic exercise can provoke an IL-6 response from muscle cells, and this finding may be associated with exercise-associated metabolic changes, adaptation to training, and possibly preconditioning individuals against myocardial ischemia-reperfusion injury [9, 11, 13]. It has further been proposed that such responses can be related to the intensity of the exercise and mediate both autocrine and paracrine benefits of training [9, 11]. While IL-6 levels are known to increase after endurance exercise, relatively little has been known on the comparisons between IL-6 and other cytokines and mediators of inflammation under such conditions. Recently, novel biomarkers of immune function, which in clinical studies have attracted interest as more specific tools to discriminate inflammation and associated disease severity, have also been developed [14–17]. Soluble urokinase-type plasminogen activator receptor (suPAR), which is expressed on various immunologically active cells, including monocytes, lymphocytes, and macrophages, is elevated in conditions involving the activation of the immune system [14–16]. Haptoglobin-hemoglobin complex (CD163) protein is an endocytic receptor for haptoglobin-hemoglobin complexes, and it is expressed solely on macrophages and monocytes [17]. It has been suggested to play a role in preventing hemoglobin-induced toxicity during physiological and pathological hemolyses [17]. Serum soluble CD163 increases in conditions with the intense involvement of macrophages possibly reflecting anti-inflammatory signaling activity. It may also contribute to protective mechanisms initiated under conditions of oxidative-radical burden [18]. However, studies on serum suPAR and CD163 under conditions of strenuous physical activity have not yet been made available.

In order to shed further light on the function of the immune system in response to acute bouts of exercise, we compared pre- and post-race values of both conventional and novel biomarkers of immune activation, including suPAR, CD163, pro-inflammatory (IL-6, IL-8, tumor necrosis factor-α [TNF-α]), anti-inflammatory (IL-10, growth factor-β [TGF-β]) cytokines and markers of muscle, heart, kidney, and liver status, among typical casual long-distance running event participants. In our sample, we chose to examine representatives from both full (42.2 km) and half-marathons. Although both distances are significant challenges for recreational runners, they still may be expected to reveal distinct differences in their associated biological and medical responses.

## Methods

We followed eight healthy male volunteers who ran either a marathon (*n* = 4) or a half-marathon (*n* = 4) race. The characteristics of the participants are summarized in Table 1. None of the subjects had any history of known diseases or reported any intake of medication due to illnesses. Except for two marathon participants, none of the subjects had previous full- or half-marathon race experience. All marathon runners were, however, fitness enthusiasts with frequent participations in different types of amateur sports activities including 10-k jogging events, cross-country skiing, or amateur tennis. In comparison, the half-marathon participants were recreational casual runners devoid of any such experience. All subjects gave their informed consent after detailed discussions on the risks, benefits, and alternatives of the experimental protocol. The protocol was approved by the Northern Osthrobothnia Hospital District (PPSHP) Institutional Review Board, Oulu, Finland, and the study was conducted according to the provisions of the Declaration of Helsinki.

**Table 1:** Main characteristics of the marathon (*n* = 4) and half-marathon (*n* = 4) runners

	Age (years)	BMI (kg/m^2^)	Finishing time (min)	Race speed (km/h)	Long-distance running experience (years)
Marathon (mean ± SD)					
	26.5 ± 15.0	22.4 ± 1.9	199.0 ± 8.8	12.7 ± 0.6	5.3 ± 6.5
Half-marathon (mean ± SD)					
	39.3 ± 13.6	23.6 ± 3.2	132.3 ± 4.5	9.6 ± 0.3	1.2 ± 0.3

*BMI* body mass index

The running trial took place in a local race with fewer than 1000 participants on a relatively flat, sea-level road course which was paired with half-marathoners doing one and marathoners doing two loops on an accurately measured course. The race commenced at 12 noon. The weather was partly cloudy with the temperature ranging from 18 to 21 °C. Relative humidity was 40–50 %, and wind speed fell between 15 and 22 km/h. During the activity, liberal amounts of fluids and exogenous carbohydrates were available ad libitum. Medical care services, as set up by experienced healthcare professionals, were available along the route throughout the race day. In addition, a medical surveillance system specifically for the study participants was established by the investigators (ON, TJ). A fully equipped medical tent was available at the finishing area. Additional medical care and blood sampling facilities were located at the Central Hospital next to the site of the running event. Medical examinations of the study participants were carried out before the race, within 2 h after finishing and 1 week after the race. Blood samples were drawn by a trained laboratory nurse on the day before and 2 days after the race after an overnight fast and a 15-min rest period prior to sampling. On the race day, blood samples were also collected 3-h post-race and before a post-race meal was provided. Serum was separated by centrifugation (1500×*g* for 10 min) and was subsequently stored at –70 °C prior to the determination of the various biomarkers. All measurements were carried out in a SFS-EN ISO/IEC 17025:2005- and SFS-EN ISO 15189:2007-accredited laboratory. Serum suPAR levels were measured using the suPARnostic enzyme-linked immunosorbent assay (ELISA) kit according to the instructions of the manufacturer (Virogates, Birkerød, Denmark). The measurements of CD163 were carried out using Quantikine human CD163 ELISA assay (R&D Systems, Abingdon Science Park, UK). The concentrations of interleukins (Il-6, IL-8, IL-10, TNF-α, TGF-β) in serum were determined using Quantikine high sensitivity ELISA kits (R&D Systems, Abingdon, UK). Serum C-reactive protein (CRP), biomarkers of muscle, heart, kidney, and liver functions, serum electrolytes, cortisol, and blood lactate were carried out using standard clinical chemical methods on Abbott Architect c8000, Abbott Architect i1000 (Abbott Diagnostics, Abbott Laboratories, Abbott Park, IL, USA), or Cobas 411 (Roche Diagnostics, Basel, Switzerland) automated clinical chemistry analyzers. Samples of EDTA-anticoagulated blood were used for the determination of blood cell counts (Sysmex automated Hematology Analyzer, Sysmex Corporation).

The values presented are reported as mean ± standard deviation (SD). Comparisons were made using paired or unpaired *t* tests, as required. Correlations between post-race values were calculated using Pearson product-moment correlation coefficients (*r*) and after an adjustment for baseline variability using percent change (pre-post) for each outcome variable. A *p* value of <0.05 was considered statistically significant. Statistical analyses were carried out on Stata statistics data analysis software (StataCorp LP, TX, USA).

## Results

The mean finishing time for the marathon was 199 ± 9 min (range 188–209 min) and for the half-marathon 132 ± 5 min (range 126–136 min) (Table 1). Post running, clinical examinations indicated pain and stiffness in the quadriceps areas in all runners, with no other clinical symptoms or signs of generalized fatigue, except for one marathon runner who needed intravenous hydration (300 mL/h) and post-race monitoring in a hospital setting. Personal feelings expressed by all participants suggested a rapid recovery, which was completed within 7 days.

**Fig 1:**
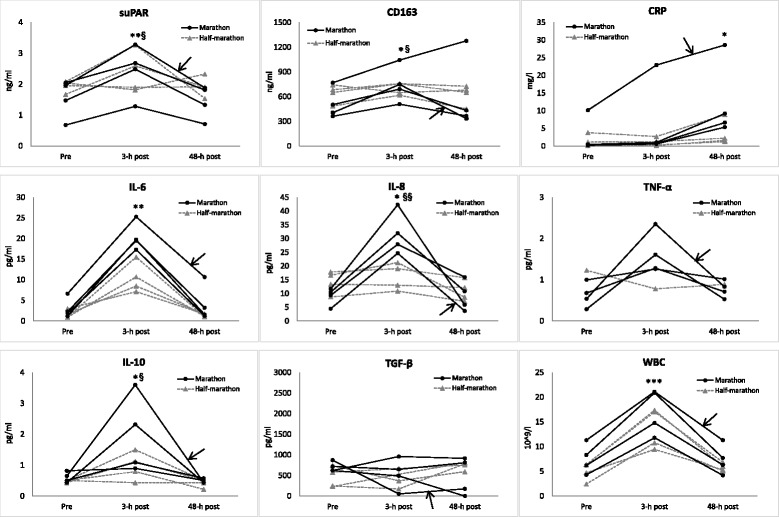
Individual changes in mediators of inflammation in recreational athletes after running either a marathon or half-marathon race. *p < 0.05, **p < 0.01, and ***p < 0.001 for comparisons between pre- and post-race values of all runners at given time points. ^§^ *p* < 0.05 and ^§§^ *p* < 0.01 for comparisons of changes in values between full- and half-marathon runners. The highest levels of suPAR, CRP, IL-6, TNF-α, and IL-10 were noted from a marathon runner who also suffered from the most severe symptoms of post-race fatigue (case indicated by *arrows*). *CD163* macrophage receptor for haptoglobin-hemoglobin complexes, *CRP* C-reactive protein, *IL* interleukin, *suPAR* soluble urokinase-type plasminogen activator receptor, *TGF-β* transforming growth factor-β, *TNF-α* tumor necrosis factor-α, *WBC* white blood cells (leukocytes)

Figure 1 summarizes the individual changes in the various biomarkers of inflammatory status in all runners. Comparison of the data at baseline and at 3-h post-race indicated significant increases in suPAR (*p* < 0.01), CD163 (*p* < 0.05), white blood cells (*p* < 0.001), pro-inflammatory cytokines, IL-6 (*p* < 0.01), and IL-8 (*p* < 0.05) whereas not in TNF-α levels (Fig. 1). Anti-inflammatory cytokine, IL-10, also showed a significant increase from baseline (*p* < 0.05) while TGF-β remained relatively stable. When comparing the data obtained from full- and half-marathon, marathon running was found to lead to more pronounced increases in serum suPAR (*p* < 0.05), CD163 (*p* < 0.05), IL-8 (*p* < 0.01), and IL-10 (*p* < 0.05) (Fig. 1). Both types of exercises increased white blood cell counts in a rather similar manner. In 48-h post-race samples, serum suPAR and cytokine markers had decreased to baseline levels, whereas an increase in serum CRP was noted especially in those marathon runners with the highest IL-6 and IL-10 levels in their preceding sample. The highest levels of serum suPAR, CRP, IL-6, TNF-α, IL-10, and cortisol were noted in the individual who also suffered from symptoms of post-race fatigue (Fig. 1).

A summary of the post-race changes in the various laboratory tests of different organ functions among marathon and half-marathon runners is shown in Table 2. The changes were most striking in biomarkers of muscle and heart injury with marked differences between the two subgroups of runners. In 3-h post-race samples, positive correlations were observed between the changes in the different mediators of inflammation (suPAR, CD163, IL-8, TNF-α, IL-10), serum uric acid and cortisol levels (Table 3). Post-race changes in mediators of inflammation also coincided with marked changes in serum myoglobin and troponin T (Fig. 1, Table 2).

**Table 2:** Post-race changes in laboratory variables, change from baseline (%)

	3-h post-race		48-h post-race	
	Marathon	Half-marathon	Marathon	Half-marathon
Myoglobin	2451	977	−14	−6
Creatine kinase	385	85	297	124
Troponin T	1744	850	98	34
Brain natriuretic peptide	226	88	93	2
Creatinine	18	34	−5	9
Aspartate aminotransferase	84	23	164	32
Alanine aminotransferase	1	3	73	10
Uric acid	30	18	0.5	3
Cortisol	87	59	−23	8
Lactate	76	0	−11	24

**Table 3:** Correlations (*r*) between changes in biomarker levels, as calculated from percent change (pre to 3 h post) for each outcome variable

	suPAR	CD163	CRP	WBC	IL-6	IL-8	TNF-α	IL-10	TGF-β	Uric acid	Cortisol	Myoglobin	Troponin T	Creatinine
CD163	0.739**													
CRP	0.358	0.561												
WBC	−0.426	−0.521	−0.352											
IL-6	0.654*	0.032	−0.001	0.117										
IL-8	0.631*	0.926****	0.570	−0.196	0.052									
TNF-α	0.729**	0.644*	0.055	−0.291	0.327	0.733**								
IL-10	0.779**	0.711**	0.139	−0.367	0.359	0.723**	0.898***							
TGF-β	−0.107	−0.417	−0.062	0.048	0.091	−0.476	−0.781**	−0.503						
Uric acid	0.782**	0.510	0.209	−0.342	0.725**	0.692*	0.103	0.649*	0.165					
Cortisol	0.632*	0.683*	0.050	−0.193	0.083	0.719**	0.877***	0.934****	−0.670*	0.243				
Myoglobin	0.245	0.369	0.634*	−0.145	0.019	0.648*	0.130	−0.241	0.249	0.589	−0.103			
Troponin T	0.455	0.364	0.712**	0.081	0.345	0.331	−0.208	−0.154	0.228	0.318	−0.105	0.690*		
Creatinine	0.469	0.188	−0.571	0.286	0.360	0.130	0.139	0.296	−0.151	0.500	0.221	0.191	0.145	
Lactate	0.495	0.544	0.705*	−0.291	−0.035	0.631*	0.189	0.401	−0.247	0.043	0.342	0.028	0.635*	−0.216

For abbreviations, see Fig. 1 caption

*p < 0.1; **p < 0.05; ***p < 0.01; ****p < 0.001

## Discussion

While physical activity in proper amounts promotes good health, abundant evidence exists also suggesting that acute bouts of vigorous exercise could trigger adverse consequences [1, 2]. Thus, there is an apparent need for additional information on the primary mechanisms of exercise-induced health effects and screening tools to prevent any possible adverse effects. Our present observations on non-elite recreational runners demonstrate distinct changes in the biomarkers of inflammation after marathon and even after only half-marathon running. There were also notable differences in the patterns of the individual responses to these two types of acute exercise bouts. While rather similar increases were noted in white blood cell counts, the response to marathon running was more pronounced in the markers of immune activation (suPAR, CD163) and the pro- (IL-8) and anti-inflammatory (IL-10) cytokines. Changes in marathon runners’ results also coincided with notable increases in biomarkers of various organ functions despite that the sample of marathon runners investigated represented individuals with more rigorous training backgrounds.

To our knowledge, the data presented here provides the first demonstration to indicate that prolonged running elevates the concentrations of soluble urokinase plasminogen activator receptor (suPAR) and the endocytic receptor for haptoglobin-hemoglobin complexes (CD163) in serum. While the former is present on various immunologically active cells, the latter is found exclusively on macrophages and monocytes. Previously, suPAR has been shown to be elevated in conditions involving severe systemic inflammation [14–16]. Studies in patients from emergency units have further indicated that suPAR levels also associate with disease prognosis. It serves as an early marker for developing critical conditions [19–21]. In an analogous manner, serum soluble CD163 has been implicated as an independent risk marker in inflammatory states [17]. Under conditions of inflammation and oxidative stress, CD163 can be released rapidly into the plasma, and it is believed to mediate an innate immune defense by sequestering hemoglobin-bound iron. This macrophage-specific receptor can also protect the individual from tissue damage by scavenging oxidative stress-induced by-products of hemoglobin [18]. It should also be noted that recent studies have emphasized an immunomodulatory role of macrophages in conferring cardioprotection [22]. Therefore, the findings that marathon running increases the levels of this macrophage-specific marker of immune activation is of note and should be considered in further trials on the relationships between inflammation and strenuous exercise.

The present observations show significant parallel increases in both pro- (IL-6, IL-8) and anti-inflammatory (IL-10) cytokines suggesting that such responses may be required for counteracting the inflammatory threat, control of tissue damage and maintenance of body function during prolonged strenuous exercise. Both IL-6 and IL-10 have been shown to be associated with stimulation of the Th2 pathway and activation of anti-inflammatory cascades, and they may also inhibit TNF-α, which is a potent mediator of tissue damage [12, 23–25]. Although the mediators of inflammation here correlated with TNF-α, the actual change in the latter did not, however, reach significance. The most marked cytokine responses were found to precede an increase in CRP, a conventional biomarker of acute phase response and of systemic inflammation. Interestingly, the highest values of CRP, suPAR, IL-6, IL-10, and serum cortisol were all found to occur in the case with the most severe post-race symptoms of fatigue, which may support the view that the status of inflammation may play a role as a determinant of performance and the stress of running a marathon. Post-race increases in serum cortisol, even despite of its notable circadian variation, also showed positive correlations with increases in the mediators of inflammation which is in line with the view suggesting an important immunomodulatory function for serum cortisol in response to exercise [26]. Current findings should be considered in the assessment of possible post-race susceptibility to infections in long-distance runners and, in particular, the relatively undertrained [27].

IL-6, which showed consistent changes as a result of both marathon and half-marathon running, has previously been shown to play a role in the regulation of acute phase response, hematopoiesis, and tissue regeneration [12]. Its systemic effects beyond the above may also include effects on the liver and the adipose tissue. Interestingly, cytokine responses, as released from muscle cells (myokines), have been recently suggested to contribute to the beneficial health effects of physical exercise and to serve as mediators of developing physical fitness [11]. There also seems to be a relationship with IL-6 responses and the intensity of the exercise [9, 11]. IL-6 has further been implicated in protective signaling against ischemic injury in the heart [13]. The primary mechanisms of interactions between IL-6 secretion from exercised muscles and its communication among muscles and other organs in conveying such protection have, however, remained largely unknown [11, 13].

An excessive release of pro-inflammatory cytokines can increase the production of reactive oxygen species and thereby serve as a trigger for oxidative stress in tissues [28]. In accordance with this view, the present data shows consistent post-race increases in white blood cells among both marathon and half-marathon participants. In marathon runners, an enhanced expression was also notable for pro-inflammatory IL-8, which is known for its ability to attract neutrophils. The changes in the mediators of inflammation coincided with an increase in serum uric acid, which due to its free radical scavenging properties can be considered a sign of an increased need of antioxidant capacity during strenuous exercise and associated free radical generation and lipid peroxidation [29, 30]. Significant correlations between acute post-race changes in the levels of serum uric acid, suPAR, and IL-6 also support a synergistic relationship between the status of inflammation and generation of oxidative stress [31]. Future studies should therefore be conducted to address the possibility of whether the use of antioxidants could influence post-race cytokine levels and the overall function of the immune system.

In the present series, the biomarkers of muscle injury used showed greater than 20-fold increases in marathon runners and greater than 10-fold increases in half-marathon runners. There was also a slight increase in serum creatinine, a marker of kidney function, which is notable in light of the fact that the release of excess muscle-derived proteins upon prolonged vigorous exercise can increase the risk of kidney damage [32–34]. Distinct changes were also noted in troponin T, a marker of myocardial damage. Previous studies have frequently reported increased serum troponin levels in some marathon finishers, although the pathogenic significance of such observations has remained unknown [5, 7, 35–37]. It is possible that enhanced myocardial wall oxidative stress together with impaired endothelial function can increase the risk of atherothrombotic events in susceptible individuals [38]. Alterations in cardiomyocyte function due to acute bouts of strenuous exercise could be mediated by activation of inflammation and oxidative stress, although the marked elevations in proBNP levels also suggest exercise-induced mechanical load in myocardium [7, 37, 39].

An obvious limitation of the present study is the small sample size. A post hoc power analysis based on the observed means and standard deviations in this present work suggested, however, a sufficient statistical power for all key findings at the recommended 0.80 level with alfa set at the conventional 0.05 level. Thus, the occurrence of type I error in the present material is unlikely. The small number of subjects may, however, have impaired our chances of reaching statistical significances in some of the comparisons. Nevertheless, the data demonstrates distinct changes in the serum levels of mediators of inflammation as a result of full- or half-marathon running performed by typical non-elite recreational athletes. These findings should be considered when evaluating the acute health effects of extreme sports with long durations. The observed changes in the normal balance of the circulating mediators of inflammation and their possible association with tissue damage, oxidative stress, and functioning of the immune system warrant further study in larger numbers.

## Conclusions

Strenuous physical activity activates immune reactions. However, few studies exist, observing exercise-induced, simultaneous changes in mediators of inflammation. In eight participants, who successfully completed the half (*n* = 4) or full (*n* = 4) marathon run, significant post-race increases occurred in levels of (1) suPAR, a biomarker of immune activation; (2) CD163, a biomarker of monocyte-macrophage activation; (3) CRP, a biomarker of acute phase response; and (4) pro- (IL-6, IL-8) and anti-inflammatory (IL-10) cytokines. While the marathon increased all parameters, half-marathon finishers showed elevated levels of IL-6 and white blood cell counts only. The highest suPAR, CRP, IL-6, TNF-α, IL-10, and cortisol levels were noted in the individual presenting with the most severe post-race fatigue. The data indicates that prolonged running increases mediators of inflammation in an exercise-dose-dependent manner which may influence post-race immune function in long-distance runners and, in particular, the relatively undertrained. The observed changes in the normal balance of the circulating mediators of inflammation and their possible association with tissue damage, oxidative stress, and post-race susceptibility to infections warrant further studies in larger populations.
